# New label-free methods for protein relative quantification applied to the investigation of an animal model of Huntington Disease

**DOI:** 10.1371/journal.pone.0238037

**Published:** 2020-09-04

**Authors:** Flora Cozzolino, Alfredo Landolfi, Ilaria Iacobucci, Vittoria Monaco, Marianna Caterino, Simona Celentano, Chiara Zuccato, Elena Cattaneo, Maria Monti

**Affiliations:** 1 Department of Chemical Sciences, University of Naples "Federico II", Naples, Italy; 2 CEINGE Advanced Biotechnologies, Naples, Italy; 3 Department of Molecular Medicine and Medical Biotechnologies, University of Naples "Federico II", Naples, Italy; 4 Department of Biosciences, University of Milan, Milan, Italy; 5 Istituto Nazionale di Genetica Molecolare "Romeo ed Enrica Invernizzi", Milan, Italy; University of Florida, UNITED STATES

## Abstract

Spectral Counts approaches (SpCs) are largely employed for the comparison of protein expression profiles in label-free (LF) differential proteomics applications. Similarly, to other comparative methods, also SpCs based approaches require a normalization procedure before Fold Changes (FC) calculation. Here, we propose new Complexity Based Normalization (CBN) methods that introduced a variable adjustment factor (f), related to the complexity of the sample, both in terms of total number of identified proteins (CBN(P)) and as total number of spectral counts (CBN(S)). Both these new methods were compared with the Normalized Spectral Abundance Factor (NSAF) and the Spectral Counts log Ratio (Rsc), by using standard protein mixtures. Finally, to test the robustness and the effectiveness of the CBNs methods, they were employed for the comparative analysis of cortical protein extract from zQ175 mouse brains, model of Huntington Disease (HD), and control animals (raw data available via ProteomeXchange with identifier PXD017471). LF data were also validated by western blot and MRM based experiments. On standard mixtures, both CBN methods showed an excellent behavior in terms of reproducibility and coefficients of variation (CVs) in comparison to the other SpCs approaches. Overall, the CBN(P) method was demonstrated to be the most reliable and sensitive in detecting small differences in protein amounts when applied to biological samples.

## Introduction

In recent years, Proteomics has gained centrality in *Omics* studies for basic and translational applications, especially for diagnostic purposes and for targeted and/or personalized medicine [1–3]. Differential proteomics approaches are attracting particular attention due to the possibility to compare protein expression profiles from multiple biological conditions, e. g. wild type vs mutant or vs pharmacologically treated, etc [4,5].

Different methodologies have been developed to carry out the qualitative-quantitative analysis of the protein content in samples using both labeled and label-free approaches. The labeling based methods employed fluorescent (e. g. Difference in Gel Electrophoresis (DIGE)) [6,7] or isotopic reagents (as for Isobaric Tag for Relative and Absolute Quantitation (iTRAQ), Stable Isotope Labeling with Amino acids in Cell culture (SILAC) and Tandem Mass Tagging (TMT) [8–12]) to differently label the two or more proteomes under investigation. These strategies provide higher levels of reproducibility because are based on the contemporaneous electrophoretic separation and/or tandem mass spectrometry (MS/MS) analysis of the samples. Despite these advantages, labeling procedures are time-consuming and very expensive for the high cost of the labeling reagents [13].

More recently, Label-Free (LF) approaches have been introduced for quantification of proteomic profiles by exploiting liquid chromatography coupled with tandem mass spectrometry (LC-MS/MS) analyses [14–16]. These procedures [17–19] represented an effective solution to overcome these drawbacks due to their reduced costs and simplified sample preparation. Nevertheless, longer analysis time and high-performance MS/MS instruments are needed.

Label-Free quantification has emerged as a consequence of the great technical advances in the development and design of high-resolution LC-MS/MS instruments [20], such as the Orbitrap mass analyzer [21]. This analytical approach relies on the measurement of two main parameters, Extracted Ion Chromatogram (XIC) and Spectral Counts (SpCs) [19,22]. Measurement of XIC is a MS1-based strategy in which the ion current associated with each peptide ion is individually considered. The relative quantification of proteins can then be obtained by evaluating the total current associated with all the peptides belonging to a specific protein in the two conditions [23]. SpCs quantification is an MS/MS-based procedure. The number of fragmentation events, (i.e. the spectral counts), measured for all the peptides belonging to the same protein in each sample are summed up resulting in the relative quantification of the protein in the different conditions [24,25].

Both XIC and SpCs methods need massive use of tandem mass spectrometry, producing a large amount of data that needs to be normalized, i.e. corrected for instrumental or uncontrollable variations before becoming suitable for quantitative purposes. In particular, specifically, designed bioinformatic tools are required to manage and process data, while appropriate normalization methods have to be used [26,27].

Normalization procedure has been assessed in multiple ways by several authors [28,29]. In general, XIC methods require steps of higher complexity such as isotopic patterns resolution, feature detection, retention time alignment, etc. Therefore, well defined and automated processing and normalization algorithms have been integrated into specifically designed software packages, as for MaxQuant [30–32].

In SpCs methods, instead, fragmentation events are crucial for both protein identification and quantification. Commonly, software workflows for SpCs quantification result in discrete, unnormalized data with normalization left to the user [33,34]. Although XIC-based methods show a greater accuracy and linear range, the access to the last generation high-resolution instruments and/or computational processing for XIC data are often prohibitive, with the consequence that SpC methods are still a valuable alternative for a large number of applications, as demonstrated by recent literature [35–38].

Here we propose a new Complexity Based Normalization (CBN) method introducing new adjustment factors in the SpC normalization formula related to the complexity of the analyzed samples in terms of total identified proteins (CBN(P)) or spectral counts (CBN(S)). Both CBN methods are compared with the multiple label-free quantification modes based on the SpC approaches, namely Ratio of Spectral Count (R_SC_) [33] and Normalized Spectra Abundance Factor (NSAF) [39].

The new proposed normalization approach was optimized on standard protein mixtures with different complexity, and then applied to the differential analysis of the proteome profiles extracted from the brain cortex of zQ175 *knock-in* mice [40], an animal model of Huntington Disease (HD), in comparison with the wild-type counterpart. The CBN(P) method was demonstrated to be the most reliable and sensitive in detecting small differences in protein amounts when applied to biological samples.

## Materials and methods

### Preparation of standard mixtures

Six standard tryptic peptide mixtures (Waters, Milford, Massachusetts, US) including Bovine Hemoglobin Chain A (HBA, Uniprot ID P01966, B. Taurus), Bovine Hemoglobin Chain B (HBB, P02070, B. Taurus), Bovine Serum Albumin (BSA, P02769, B. Taurus), yeast Enolase (ENO, P00924, S. Cerevisiae), yeast Alcohol Dehydrogenase (ADH, P00330, S. Cerevisiae) and rabbit Glycogen Phosphorylase (PYG, P00489, O. Cuniculus) were dissolved in 0.2% formic acid (FA) and spiked into an *E*. *Coli* total tryptic digest (Waters, Milford, Massachusetts, US) solution, so that final concentration of the digest reaches 67 ng/μL. In all mixtures obtained and indicated with A-E letters, HBA, HBB, BSA and PYG were always added in a fixed amount, while only ENO and ADH were added in variable amounts ranging from 1/25 to 5 times the fixed standards (i.e. 0.94–23.5ng/μl for ENO and 0.76–19 ng/μl for ADH). The obtained standard mixtures have been employed for the optimization of mass spectrometry analyses and for setting quantification methods.

### The zQ175 mouse model

All protocols involving animals were carried out in accordance with institutional guidelines in compliance with Italian law (D. Lgs no. 2014/26, implementation of the 2010/63/UE) and authorization n.324/2015-PR issued May 6, 2015, by Ministry of Health. The Ethics Committee of the University of Milano approved studies in mice (Ethical Approval 74/13; Ethical Approval 74/14). JAX stock number was B6J.129S1-Htt tm1Mfc /190ChdiJ. For biochemical analyses, animals were euthanized by dislocation and all efforts were made to minimize suffering. Genotyping of the zQ175 (C57BL/6J) mouse colony was performed by PCR of genomic DNA obtained from tail samples (Nucleo Spin Tissue, Macherey-Nagel, catalog 740952.250) at weaning. CAG repeats of zQ175 mice were sized by using the following PCR primers: forward: CATTCATTGCCTTGCTGCTAAG; reverse: CTGAAACGACTTGAGCGACTC. Cycling conditions were 94°C for 10 minutes, 30 cycles × (96°C for 30 seconds, 57°C for 30 seconds, 72°C for 30 seconds), 72°C for 7 minutes.

### Preparation of total protein lysates from mouse cortical tissues

For proteomics and biochemical analyses, animals were euthanized by dislocation and the brains were rapidly removed and the cerebral cortices were immediately excised from the brain, frozen in liquid nitrogen and smashed. Specifically, cortical tissues from three symptomatic homozygous zQ175 mutant and three wild type (WT) mice at 50 weeks of age were used. The samples were then lysed by combining chemical and mechanical methods. Cortical tissues were crushed and repeatedly pipetted in the lysis buffer (50 mM Tris-HCl pH 7.4, 150 mM NaCl, 0.1% v/v Sodium Dodecyl Sulphate (SDS), 1 mM Phenylmethylsulfonyl fluoride (PMSF), 1% v/v Nonidet P-40 (NP40), protease inhibitors EDTA-free), then passed ten times each through a syringe. Following these mechanical processes, they were put on a stirring wheel, (20 minutes at 4°C), and then centrifuged, (16,100 x g, 30 minutes, 4°C). After the lysis procedure, supernatants from all samples were collected and quantified according to Bradford protein assay and 50 μg from each were used for the Differential Proteomics experiment and Western Blot assays.

### SDS-PAGE and *in situ* hydrolysis

50μg from each sample were suspended in Laemmli buffer containing 100 mM dithiothreitol (DTT), boiled for 10 minutes and loaded on a 20x20 cm 4% - 15% bis-acrylamide gradient gel. After SDS-PAGE separation, the gel was stained with Coomassie Blue Brilliant and each lane was excised in 29 slices by using the same cutting scheme. All gel slices were *in situ* hydrolyzed as previously reported [41]. Finally, the peptide mixtures were vacuum-dried and resuspended in 0.2% formic acid (FA) solution to be analyzed by LC-MS/MS.

### LC-MS/MS analyses

Quantitative data were collected on an LTQ Orbitrap XL (ThermoScientific, Waltham, MA) coupled to a nanoLC system (nanoEasy II). All peptide mixtures were analyzed using the same chromatographic conditions, i.e. 3 microliters of each sample were fractionated onto a C18 capillary reverse-phase column (100 mm, 75 μm, 5 μm) working at 250 nl/min flow rate, using a linear gradient of eluent B (0.2% formic acid in 95% acetonitrile) in A (0.2% formic acid and 2%acetonitrile in MilliQ water) from 5% to 40% in 80 minutes was run. MS/MS analyses were performed using Data-Dependent Acquisition (DDA) mode: one MS scan (mass range from 400 to 1800 m/z) was followed by MS/MS scans of the five most abundant ions in each MS scan, applying a dynamic exclusion window of 40 seconds. All samples were run in duplicates.

### Protein identification and quantification

Raw data obtained from nanoLC-MS/MS were analyzed with MaxQuant 1.5.2 integrated with the Andromeda search engine [42]. To that end, an appropriate Fasta file was generated by downloading from UniProt and subsequently merging amino acid sequences of both standard proteins and whole *E*. *Coli* proteome.

The selected parameters for protein identification were the following: minimum 2 peptides, at least 1 unique; variable modifications allowed were methionine oxidation and pyroglutamate formation on N-terminal glutamine; accuracy for the first search was set to 10 ppm, then lowered to 5 ppm in the main search; 0.01 FDR was used, with a reverse database for decoy; retention time alignment and second peptides search functions were allowed. Protein quantification has been performed only using unique unmodified peptides.

As output, MaxQuant resulted in discrete spectral counts, which were then normalized by using NSAF, R_SC_, or CBNs approaches.

As concerns data collected for the Differential Proteomics analysis on zQ175 mice, following MaxQuant analysis data, each couple of technical duplicate was mediated for every protein, obtaining a set of 6 data (3 for WT and 3 for zQ175). These values were employed for manual normalization according to the above mentioned SpCs methods.

### Western blot and densitometric analyses

Mice brains total protein extracts were separated by SDS-PAGE and then transferred onto nitrocellulose membranes (Bio-rad, Hercules, California, US). Membranes were blocked with 5% non-fat milk and then incubated with the following antibodies: rabbit polyclonal anti-hnRNPH (Abcam, ab154894), rabbit polyclonal anti-SERPIN B6 (Abcam, ab233229; Proteintech, 14962-1-AP), rabbit polyclonal anti-IRGM (Abcam, ab118569), rabbit polyclonal anti-UQCRQ (Proteintech, 14975-1-AP), rabbit monoclonal anti-HOMER1 (Abcam, ab184955), rabbit monoclonal anti-HTT (Cell Signalling Technology, mAb#5656), rabbit polyclonal anti-OSBPL2 (Proteintech, 14751-1-AP), mouse monoclonal anti-β-Actin (Origene, TA811000) and mouse monoclonal anti-Vinculin (Sigma-Aldrich, V9131). Membranes were incubated with horseradish peroxidase-conjugated secondary antibody (1:5000 for mouse host antibodies and 1:10000 for rabbit host antibodies) for 45 minutes at room temperature and the signals were detected by enhanced chemiluminescence (ECL) detection system (Thermo Fisher Scientific, Inc., Waltham, MA).

For densitometric analyses, the software Quantity One 4.6.8 (Bio-Rad, Hercules, California, US) has been employed; all protein band intensities were normalized with the corresponding β–actin signal, except HTT, whose intensities were normalized on vinculin signal.

### Multiple-Reaction Monitoring (MRM) analyses

As additional validation method multiple-reaction monitoring (MRM) approach was employed. 50 μg of cell lysates obtained as described above were digested by trypsin onto S-Trap filters, according to the manufacturer protocol (Protifi, Huntington, NY). The three biological replicates of zQ175 and wild-type mice were pooled respectively, and HTT, SERPIN B6, HOMER1, OSPBL2, SAMM50, APO-A4, CAMK2A, STIP1, RIC8A, ATP2A2 peptide transitions were monitored with a Xevo-TQS mass spectrometer coupled to a nanoAcquity UHPLC (Waters, Milford, MA, US) equipped with IonKey CHIP interface. Peptide mixtures were separated on peptide BEH C18 130Ǻ, 1.7μm, 150 μm x 50mm, iKey by using a linear gradient of eluent B (95% acetonitrile LC-MS grade (Sigma Aldrich, St. Louis, Missouri, US), 0.2% formic acid (Sigma Aldrich, St. Louis, Missouri, US)) from 7% to 95% over 115 minutes working at a flow rate of 3μl/min. Raw data were processed with Skyline v20.1.0.155 (MacCoss Lab Software, Dept of Genome Sciences, UW) and the total area of each peptide transition was used for the relative quantification of the specific proteins. For each protein, at least two prototypic peptides were selected and at least two transitions for each parent ion were monitored, as *in silico* predicted by using Skyline. Proteins were not monitored all together but in three different runs, both for wild type and zQ175: run A, included HTT, HOMER1, RIC8A; run B, ATP2A2, APO-A4, OSBPL2; run C, SAMM50, SERPIN B6, CAMK2A, STIP1 and in each run ACTIN was monitored as the internal standard. Each run was analyzed in duplicate and the total area of each peptide transitions was used for the relative quantification of the specific proteins, normalized for actin FC.

### Statistical analyses

Multiexperiment Viewer v4.9.0 [43], (MeV) was employed to perform statistical analysis of MaxQuant output. Western blots results were evaluated by univariate statistical analysis using GraphPad Prism 8.0, and the results are presented as the mean ± standard deviation (SD) by three biological replicates. The statistical significance of the observed difference in western blot analysis was determined by parametric (Welch’s t) or non-parametric (Mann–Whitney test) tests when data failed the Shapiro–Wilk normality test. Mass spectrometry data (LF) statistical significance was determined by the unpaired Student’s t-test and differences were considered statistically significant at Benjamini-Hochberg corrected p-value (FDR)<0.05 [44].

### Functional over-representation analysis

STRING database (https://string-db.org/) [45] (STRING v11: Protein-Protein Association Networks With Increased Coverage, Supporting Functional Discovery in Genome-Wide Experimental Datasets) was used to perform a pathways enrichment analysis on differentially expressed proteins in mouse brains identified in each method (NSAF, Rsc, CBN(P), CBN(S)).

## Results

### Development of a new method for Spectral Counts normalization

A new mathematic method, called Complexity Based Normalization (CBN), was developed for the normalization of Spectral Counts data in differential proteomics experiments to calculate the relative Fold Change for each protein between two experimental conditions. The new formula (1) employed for SpCs normalization is reported as follows in comparison with the Normalized Spectra Abundance Factor (NSAF) (2) and the Spectral Counts log Ratio (R_SC_) (3) methods.

$${CBN}_{x,M}=\frac{S_{x,M}}{t_{M}}+f$$ (1)

$${NSAF}_{x,M}=\frac{\frac{S_{x,M}}{L_{x}}}{\sum_{i=1}^{P}\frac{S_{i,M}}{L_{i}}}$$ (2)

$$R_{{SC}_{x,M}}={log}_{2}\left(\frac{S_{x,M}+f}{t_{M}-S_{x,M}+f}\right)$$ (3)

In CBN methods (1), S_x,M_ are the Spectral Counts of protein x in mixture M, t_M_ are the total spectral counts of mixture M, f is the complexity-based adjustment factor. In addition to common variables, in NSAF method (2) L_i_ represents the amino acid length of "i" protein, while P is the total number of proteins identified in the analysis. Finally, in the R_SC_ method (3) f is a fixed adjustment factor = 0.5.

The output of these formulas consists of a numeric value representing the quantity of a specific protein in each mixture that is used to calculate the relative Fold Change (FC). Both R_SC_ and CBN require the use of an adjustment factor "f" to avoid the presence of missing values. In the R_SC_ method, Beissbarth et al. [46] have fixed the f value constant to 0.5. For both CBN methods we propose the employment of a variable adjustment factor according to sample complexity. Specifically, the CBN formula has been developed with f = 1/P (CNB(P)), where P is the number of proteins occurring in the MaxQuant output data or with f = 1/t (CBN(S)), where t is the sum of all spectral counts associated to all proteins in all mixtures. As a consequence of these definitions, the adjustment factor becomes complexity-based, since the P and t values are both related to the total number of proteins in the sample.

### Setting up the normalization methods on *E*. *Coli* proteome standard sample

The newly developed CBN formulas were evaluated in comparison with existing normalization methods in differential proteomics experiments using the label-free procedure applied to the *E*. *Coli* proteome. Commercial tryptic digests from six different proteins (HBA, HBB, and BSA from *B*. *Taurus*, ENO and ADH from *S*. *Cerevisiae* and PYG, from *O*. *Cuniculus*) were spiked into a fixed matrix consisting of a constant amount of *E*. *Coli* total proteins tryptic digest, to mimic a real sample environment. Five mixtures (A, B, C, D, E) were prepared as described above, in which only ADH and ENO tryptic peptides were added in different amounts. Each mixture was analyzed by nanoLC-MS/MS in duplicates and the raw data processed by using MaxQuant. Following MaxQuant analysis, a set of unnormalized SpCs data was obtained and used to test the different normalization methodologies, NSAF, Rsc, and the newly developed CBNs, either in terms of total identified proteins (CBN(P)) or spectral counts (CBN(S)).

A total of 409 proteins (P), including the six spiked standards were identified in all samples by MaxQuant analysis, together with 17837 total spectral counts (t) (S1 Table). The P and t experimental values were then introduced in the CBN formulas for the computation of the f factor.

**Fig 1:**
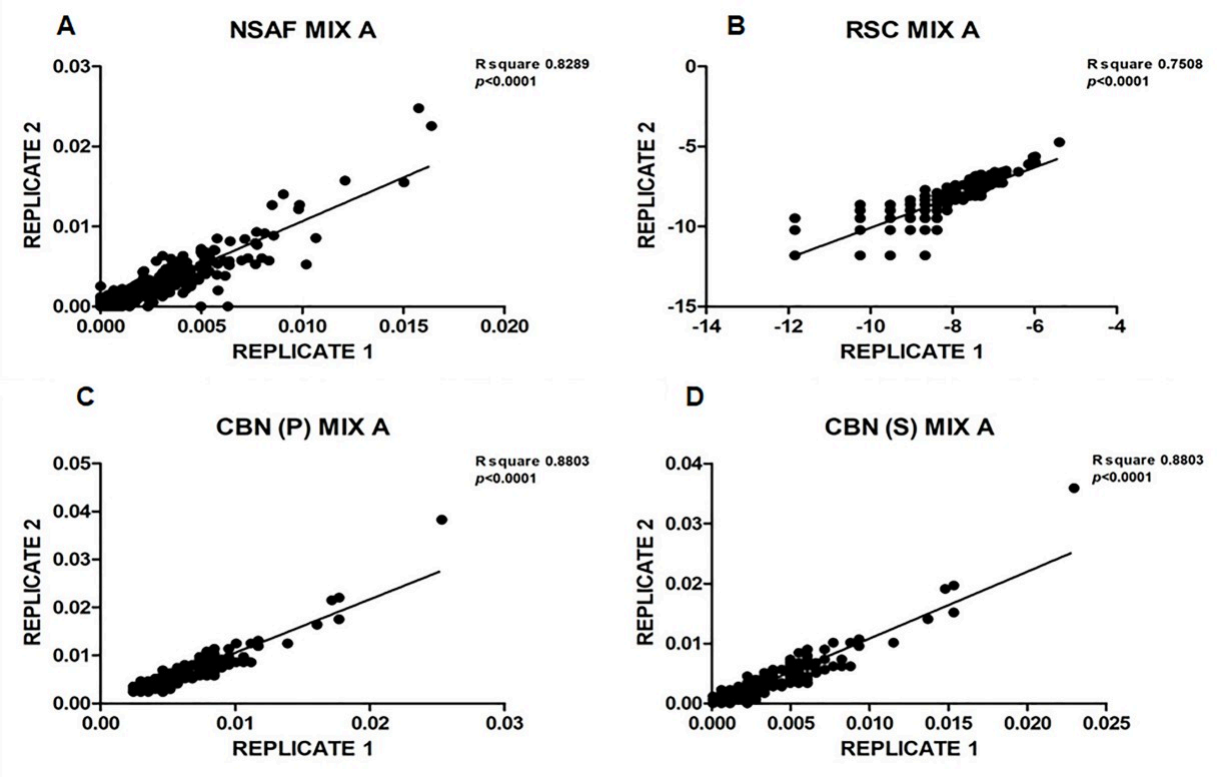
Scatter plots of the quantitative measurements of all the *E*. *Coli* proteins. Scatter plots of the quantitative measurements of all the *E*. *Coli* proteins identified in the two replicates of mixture A by the four different normalization methods used, i.e. NSAF (panel A), R_SC_ (panel B), CBN(P) with f = 1/P (panel C) and CBN(S) with f = 1/t (panel D). On the X-axis is reported the normalized Spectral Counts of replicate 1, and on the Y-axis the normalized Spectral Counts of replicate 2.

As a first step, the reproducibility of the different approaches was tested making use of the two technical replicates prepared and analyzed for each mixture. As an example, Fig 1A–1D shows the corresponding scatter plots concerning the quantitative measurements of all proteins identified in the two replicates of mixture A by the four different normalization methods used, i.e. NSAF, R_SC_, CBN(P) with f = 1/P and CBN(S) with f = 1/t.

When the data produced by the applied method were perfectly reproducible between the two technical replicates, the scatter plot should result in a perfectly linear behavior (R^2^ = 1) and, most importantly, should have a unitary slope.

As showed in Fig 1A–1D, a very poor correlation occurred when data were elaborated by NSAF and Rsc in comparison to CBN methods, with a high number of points largely scattered, therefore suggesting a lower level of reproducibility. Linear regression best-fit values are summarized in S2 Table. It should be underlined that the scatter plots associated with the two CBN methods showed quite similar shape although the value of the adjustment factor was greatly different. Furthermore, as shown in panel 1B, a large scattering of data that could not be accommodated in a linear behavior was observed in the R_SC_ normalization method.

**Fig 2:**
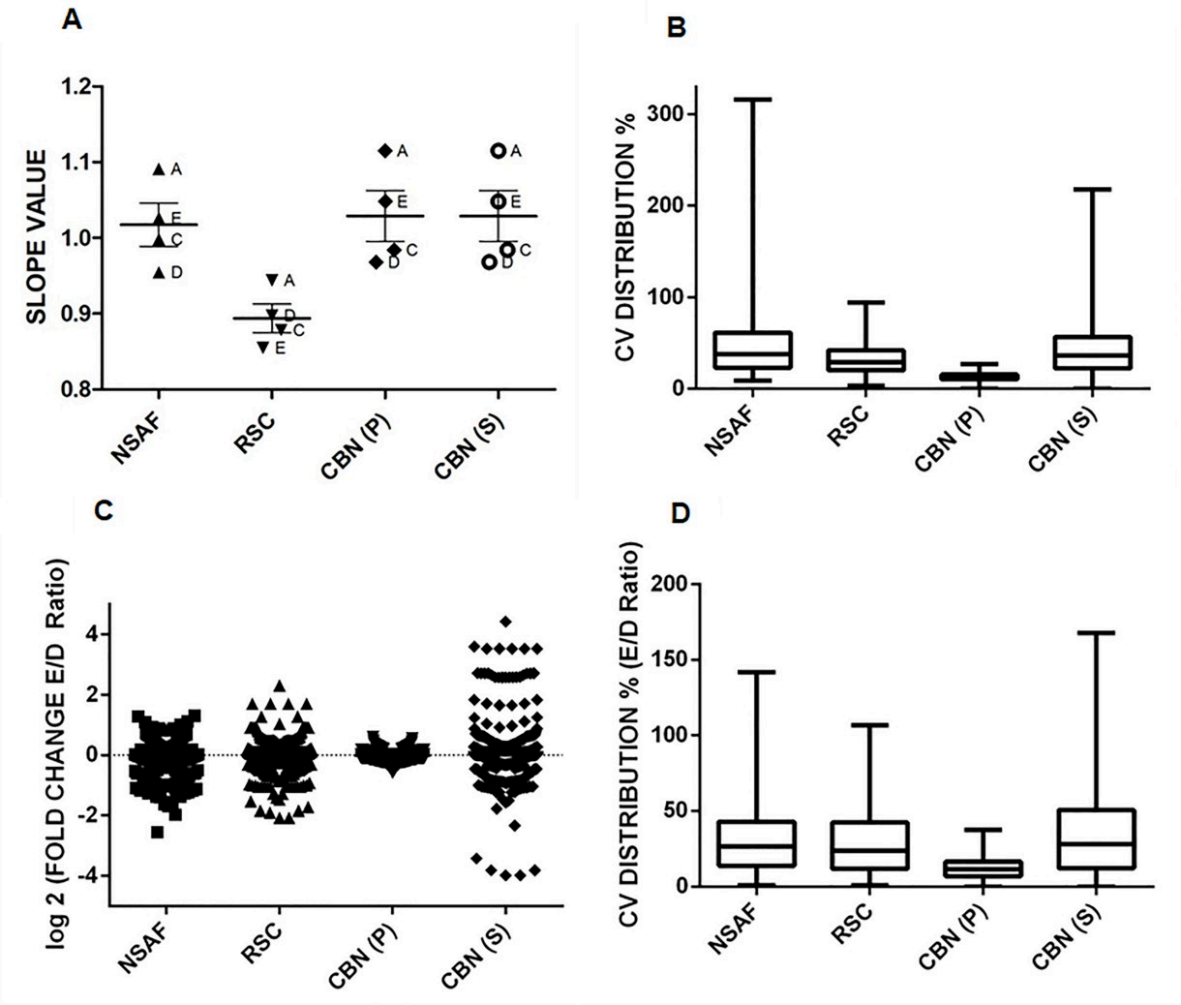
Comparative analysis of all spectral counts normalization methods applied to *E*. *Coli* proteome. (A) Median values for the best fitting slopes calculated for each pair of technical replicates for all samples analyzed (except mixture B) with each normalization method. (B) Coefficient of variation (CV) for the evaluation of data dispersion for all the normalization methods used in the analysis of *E*. *Coli* proteome. (C) Representation of the logarithmic Fold Change distribution around the theoretical value indicated by the point line for all normalization methods. (D) Box plots of the CV for the fold change in the mix E / mix D pair, calculated on all the four possible pairs of the technical replicates. In panels B and D the box and whisker extremes represent 25–75%.

Fig 2A reports the slopes calculated for the best fitting lines in each pair of technical replicates analyzed with the four methods for all samples except mixture B, since it showed a very low reproducibility in all methods (S1 Fig), suggesting the occurrence of technical problems in the LC-MS/MS analysis. Findings reported in Fig 2A showed that all the SpCs methods displayed an acceptable behavior except for R_SC,_ whose median value was much lower than the expected value (i.e. 1) suggesting that a fixed adjustment factor "f" strongly affects the median value. The reproducibility of each method was also evaluated by calculating the coefficient of variation (CV) in the quantification of *E*. *Coli* proteins throughout the ten LC-MS/MS runs in which the amount of *E*. *Coli* tryptic peptides was constant (Fig 2B). The R_SC_ method showed a very good behavior with a low coefficient of variation whereas the NSAF displayed a greater dispersion of data and a very high CV value. This is very likely due to the absence of any adjustment factor in this method making NSAF very susceptible to quantitative biases caused by the absence of data. As showed, CBN(S) performed well, showing a CV dispersion intermediate between NSAF and Rsc. The best result occurred in CBN(P), which presented the lowest CV values contained in the narrowest dispersion window, suggesting it as the most precise among the investigated methods.

These preliminary data strengthened our hypothesis that differences in Fold Change values are due to the different sizes of the adjustment factor, since the shape of the data is identical.

The normalization methods were then tested for their ability to correctly assess protein quantification when two different sets of data were compared. Therefore, the five mixtures and the corresponding replicates were compared by two according to the scheme: A1/B1, A2/B1, A1/B2, A2/B2, etc., and the Fold Change (FC) values of all proteins in each pair were calculated. As the amount of *E*. *Coli* proteome was constant in all samples, the expecting Folding Change value for each protein should have been equal to 1 (Log_2_ (FC) = 0, Fig 2C).

For each pair, the mean value of the Fold Change and its associated CV was calculated. As an example, in Fig 2D the data related to the four possible combinations of mixture E/mixture D pair were reported. Inspection of the results showed a very regular distribution of data points for the CBN(P) method, with more than 75% of values having an associated CV of 25% or less. NSAF and Rsc displayed a slightly more dispersed distribution of Fold Change values, while CBN(S) showed the highest dispersion of data.

Then, we moved to evaluate the statistical relevance of proposed methods, through the calculation of False Discovery Rate (FDR). An unpaired Student's t-test was performed to extrapolate the *E*. *Coli* statistically significant Differentially Expressed Proteins (DEPs): the latter represented the pool of false positives, since no variations were expected in *E*. *Coli* proteins. The FDRs, calculated as the percentage of false positives of total identified proteins, resulted in 5.1% for Rsc, 6.1% for both CBNs, and 7.6% for NSAF. These values are very close to the reference one (5%), indicating a high reliability of all methods, excepted NSAF, which showed the highest value of false positives.

Furthermore, for each method the values of Fold Change cutoffs were estimated, treating the standard mixtures A—C and D—E as a couple of samples to be compared (A and C vs D and E).

The FC cutoffs were calculated for each method by evaluating the dispersion of FCs of the unchangeable proteins of *E*. *Coli* proteome, considered not statistically significant according to unpaired Student's t-test (p<0.05). The FC thresholds were defined by the lower Q1-(IQR*1.5) and upper Q3+(IQR*1.5) extremes, where Q1, Q3 represented 25 and 75 percentiles respectively, and IQR is defined as the inter-quartile distance. The obtained FC cutoffs were summarized in Table 1:

**Table 1:** Methods fold changes cutoffs.

METHOD	Lower FC value	Upper FC value
**CBN(P)**	0.79	1.20
**CBN(S)**	0.49	1.53
**NSAF**	0.45	1.60
**RSC**	0.51	1.51

Fold Changes cutoffs calculated for each method are reported, indicating upper and lower significant values.

These findings showed very similar results for CBN(S), Rsc, and NSAF, while CBN(P) performed quite differently. In particular, its narrowest cutoffs again confirmed that CBN(P) is the most reliable method in the detection of slight differences in protein expression levels (low FCs), which in biological samples might be relevant.

### Comparison of normalization methods for the quantification of the six standard proteins spiked within the *E*. *Coli* proteome

The different normalization methods were then evaluated in the quantification of the Fold Changes of the six standard proteins spiked within the *E*. *Coli* proteome.

The Fold Change values were determined by all methods for the six standard proteins spiked within the *E*. *Coli* proteome in all mixtures (S3 Table).

**Fig 3:**
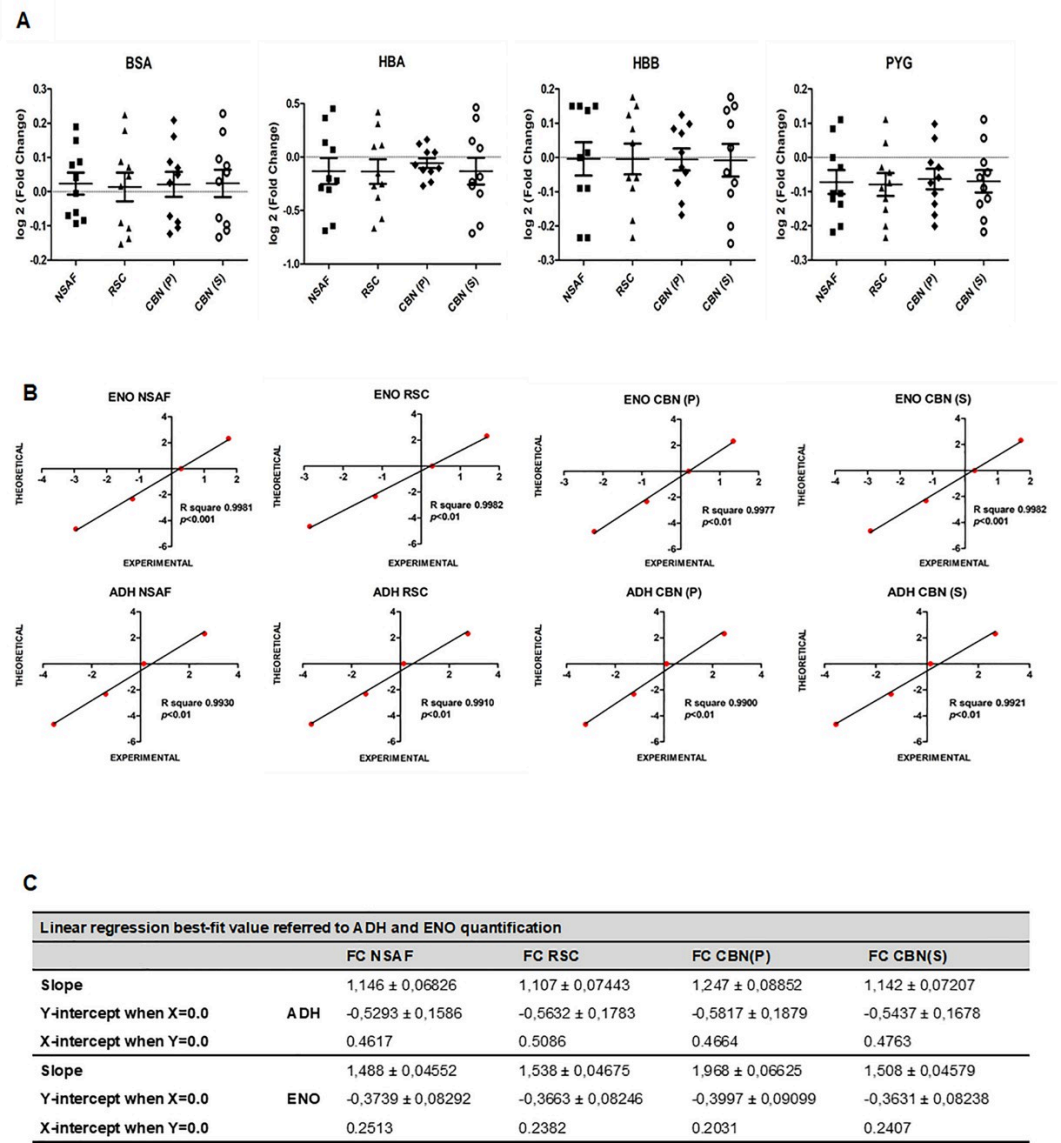
Fold change analysis comparing all spectral counts normalization methods. (A) The mean Fold Change values for unchangeable proteins (BSA, HBA, HBB, and PYG) within the ten different pairs of mixtures calculated by the four normalization methods. The theoretical values are 0 and it is indicated by a point line. (B) Comparison of the experimental and theoretical Fold Change values for the relative quantification of ENO and ADH obtained by using the SpC-based methods and reported in the logarithmic scale. (C) Table reporting linear regression best-fit values for all ENO and ADH linear regressions.

The performances of the different normalization methods were evaluated for the quantification of BSA, HBA, HBB, and PYG proteins, whose concentration was constant in all mixtures, and for quantification of ENO and ADH, whose amount changed in the samples. Fig 3A reports plots showing the distribution of the logarithmic Fold Change for unchangeable proteins obtained with the four normalization methods. All methods displayed similar results indicating a general high accuracy in the FC measurements, as mainly demonstrated in BSA and HBB, where the mean values agree to the true value (FC = 0). In HBA the best fit occurred in CBN(P) method, confirming very good performances in terms of accuracy and precision. However, almost all measured values scattered within 0.3 logarithmic units from the theoretical values, corresponding to an absolute Fold Change of 1.2, which is reasonably considered as an unchanged amount of protein.

The linear correlations between experimental and theoretical values obtained for all different normalization methods were investigated for ENO and ADH, whose concentrations were changeable (Fig 3B). Very similar correlation values were obtained for both proteins in the calculation of relative abundance determined by NSAF, Rsc and CBNs, suggesting that all methods are equally able to correctly evaluate differences in the amount of proteins. For ENO, linear correlations were confirmed also decreasing 10 times the concentration of the lowest point reported in Fig 3B (data not shown), confirming a good behavior for all methods.

In conclusion, the quantitative measurement using standard mixtures demonstrated that both the newly proposed CBN methods resulted reliable in the estimation of all expected FCs, either when the protein amount changed or when it was constant, for which they showed a very good adherence to theoretical data even in a complex mixture.

### Application of CBN methods to the differential proteomics analysis of samples from cerebral cortex from Huntington’s Disease mice

Once the effectiveness of the newly developed SpC-based normalization methods was defined, the two CBN approaches were challenged with the analysis of more complex samples, in which the expression level of a large number of proteins is expected to significantly change. A label-free differential proteomics experiment was performed on the brain cortex proteome from mice affected by Huntington’s Disease (HD) (homozygous mutant HTT *knock in* line zQ175) [40] and corresponding control samples (WT).

Equal amounts of total protein extracts from three wild type and three mutant mice cortices were separated by SDS-PAGE (S2 Fig) and each of the six lanes was cut in 29 slices that were *in situ* hydrolyzed with trypsin. The corresponding 174 peptide mixtures were then analyzed by nanoLC-MS/MS in duplicates.

**Fig 4:**
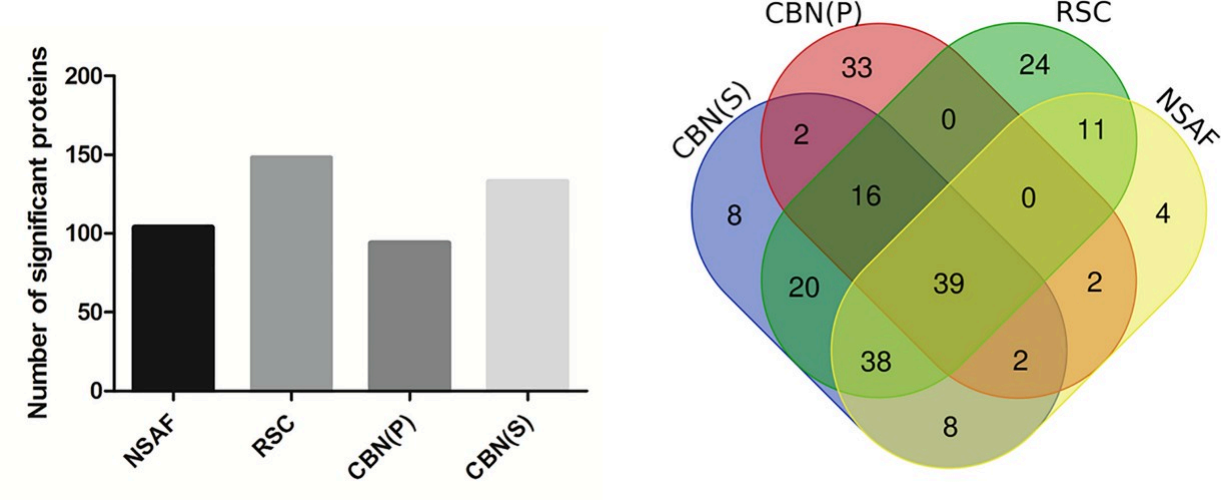
Visualization of identified proteins with all methods in the WT and HD mouse model. (A) The histogram shows for each method the number of proteins that appeared to be both differentially expressed and statistically significant. (B) Venn diagram referred to all proteins. The central area represents the proteins common to all methods.

The resulting set of 348 LC-MS/MS raw data (available via ProteomeXchange with identifier PXD017471) was processed by MaxQuant for protein identification and quantification (S4 Table). The set of unnormalized SpCs data was then elaborated by the newly developed CBN methods (CBN(P) and CBN(S)). The other methods (NSAF and Rsc) were used for comparison. The lists of proteins were then filtered according to their differential expression and statistical significance (FDR<5%) by using the Multi experiment Viewer, as described above. Statistically significant up- and down-regulated proteins, identified by each method according to FC cut off values previously calculated, were reported in the histogram in Fig 4A. It is surprising to note that the two CBN methods led to the identification of a quite different number of statistically significant proteins. In CBN(S) as well as Rsc, it was nearly 140, whereas the CBN(P) as well as the NSAF approach led to the identification of only about a hundred of significant proteins.

In particular, the specific and shared proteins were highlighted in a Venn diagram (Fig 4B). Details of statistically significant identified proteins were summarized in S5 Table.

The reliability of the proposed normalization methods was further confirmed by quantification of some selected proteins by two independent methodologies, i.e. western blot analyses and Multiple Reaction Monitoring (MRM) mass spectrometry analysis.

**Fig 5:**
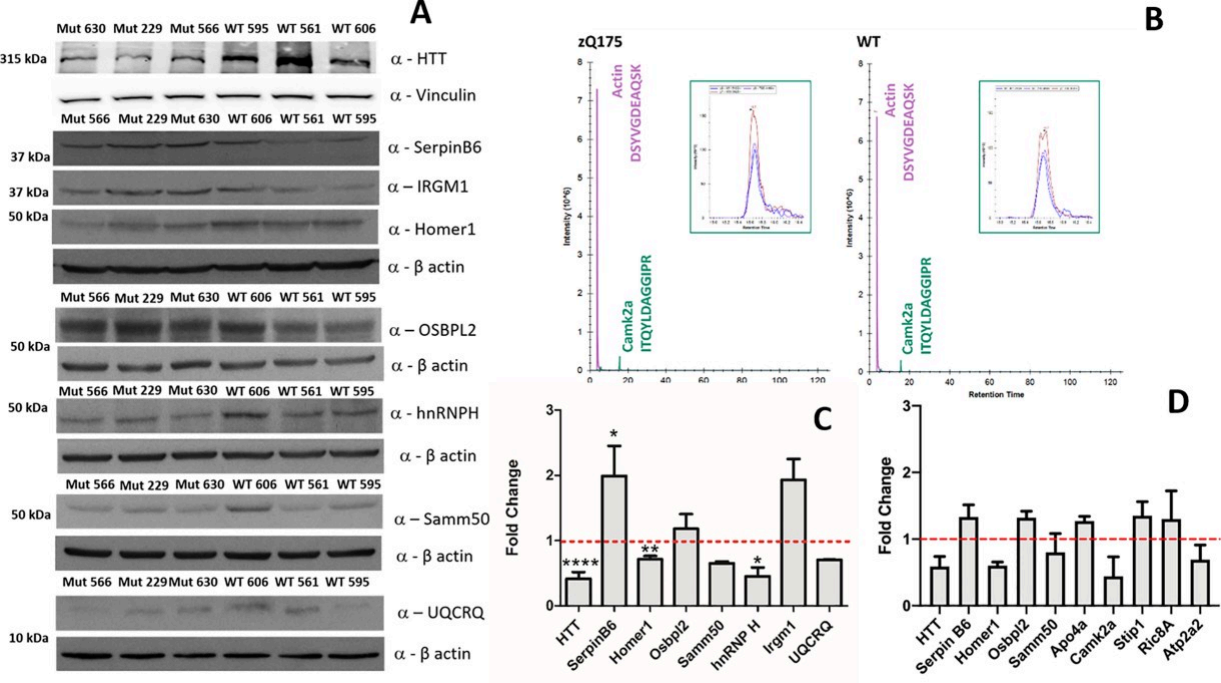
Validation of a selected group of proteins differentially expressed in WT and HD mouse model. (A) Western blot assays performed on total protein extracts from three mutant (zQ175(1), zQ175(2), zQ175(3)), and three wild-type (wt(1), wt(2), wt(3)) mice with antibodies against the selected proteins. β-actin was used for normalization. (B) MRM superimposed traces of transitions of one of CAMK2A proteotypic peptide reported for zQ175 (left panel) and WT (right panel) together with one ACTIN peptide transition. (C) Densitometric analysis of data from the western blot of panel A. The indicated values in the graph represent the percentage of arbitrary units compared to WT to which 100% was assigned. Results are represented as the as mean ± SD (standard deviation). The statistical significance was evaluated by parametric (Welch’s) or non-parametric (Mann-Whitney) tests when data failed the Shapiro–Wilk normality test. * p < 0.05, ** p < 0.01 *** p < 0.001, **** p < 0.0001. (D) Fold Change measured by MRM analysis of pooled HD and WT samples, respectively.

In western blot assays (Fig 5A), the expression levels of IRGM1, OSBPL2 and SERPIN B6, which were up-regulated in HD mice and hnRNP H, HTT, SAMM50, UQCRQ and HOMER1, whose expression was instead decreased in HD mice were evaluated on total protein extracts of three WT and three HD mice (the same samples employed for the proteomic experiment), in duplicate.

Upon the densitometric analysis, each sample was normalized with the intensity of β-actin developed on the same membrane, except HTT normalized with the intensity of vinculin. The normalized intensities were then evaluated by univariate statistical analysis and the results summarized in Table 2 and graphically reported in Fig 5C.

**Table 2:** Summary of proteins FCs validated by western blot and/or MRM.

Protein	FC MRM	FC WB	FC CBN(S)	FC CBN(P)	FC RSC	FC NSAF
**HTT**	0.59	0.42	0.08	0.59	0.14	/
**CAMK2A**	0.44	/	/	0.67	/	/
**SAMM50**	0.80	0.66	0.44	0.69	0.47	/
**ATP2A2**	0.69	/	/	0.69	/	/
**HOMER1**	0.60	0.72	0.45	0.71	0.45	/
**IRGM1**	/	1.93	19.73	1.22	4.09	UP
**APO-A4**	1.27	/	/	1.29	/	/
**RIC8A**	1.30	/	4.46	1.30	/	/
**STIP1**	1.35	/	2.38	1.32	2.11	2.50
**OSBPL2**	1.32	1.18	6.37	/	2.92	/
**SERPIN B6**	1.33	1.99	2.55	/	2.02	2.75
**HNRNP H**	/	0.46	0.30	/	/	/
**UQCRQ**	/	0.71	0.36	/	/	/

In the table proteins selected for validation and their FC values calculated by LF, MRM, and WB methods are reported.

Since in several cases the differences in expression levels were about 20–30%, the western blot technique might not be enough reliable in detections on a restricted number of replicates.

Therefore, we integrated validation experiments by quantifying some proteins by using a tandem mass spectrometry methodology: the Multiple Reaction Monitoring (MRM). HTT, SERPIN B6, HOMER1, OSBPL2, and SAMM50 were also monitored by MRM, together with other proteins, APO-A4, CAMK2A, STIP1, RIC8A and ATP2A2, whose antibodies were not available or not efficient in western blot analyses. ACTIN was monitored too and employed as an internal standard for normalization procedure.

In detail, three zQ175 and WT cortex brain samples (the same samples employed for the proteomic experiment) were pooled respectively, and few micrograms were employed for a shotgun proteomics experiment by using the MRM targeted approach. Peptide mixtures were separated by nanoLC-MS/MS in duplicate and two or more transitions of at least two proteotypic peptides were monitored for each of the above proteins and employed for relative protein quantification. Areas of monitored transitions (Fig 5B, S6 Table) were measured, mediated among transitions, and all peptides belonging to the same protein, employed to calculate FC, which were normalized with FC measured for ACTIN in each couple of runs. FC results are summarized in Table 2 and graphically reported in Fig 5D.

A strong agreement among all measured FCs emerged by comparing the results obtained by western blot and MRM, confirming the reliability in the validation of both methods. Moreover, for all proteins, the variation trends detected by LF approaches were confirmed by both methods (Table 2) also in the detection of slight FCs.

In detail, levels of HTT protein are significantly reduced in HD mice compared to controls, according to previously observed [47]. This protein was identified by Rsc and CBN approaches as statistically significant, although the three methods measured very different FC values: CBN(S) FC = 0.08, CBN(P) FC = 0.59, Rsc FC = 0.14. By comparing these values with those obtained by western blot (0.42) and MRM (0.59), the FC measured by CBN(P) was the only perfectly in agreement with both values. This finding was not a random or a sporadic event, but it was recurrent in all proteins, from those quantified also in other methods (HTT, SAMM50, HOMER1, IRGM1, RIC8A, STIP1, OSBPL2, UQCRQ) to those statistically significant only for CBN(P) (CAMK2A, APO-A4, ATP2A2) (Table 2).

hnRNP H and UQCRQ are proteins identified as statistically significant only by CBN(S), while SERPIN B6 varied significantly for all methods excepted CBN(P). hnRNP H and SERPIN B6 were endorsed by western blot assays, founding their densitometric mean values statistically significant. Surprisingly, FC values calculated by CBN(S) for these proteins strongly agreed with those obtained from the other methods. Altogether these data confirm that the quantification of results obtained by normalization of the differential proteomic data performed by the CBN methods is reliable, although CBN(P) was confirmed to be the best in terms of precision and reliability in comparison with CBN(S) and the other investigated methods.

The results of the differential proteomics experiments were then evaluated on a functional basis by gathering the differentially expressed proteins within biological functional networks. A bioinformatic analysis involving all the differentially expressed proteins identified by all methods were carried out using KEGG pathways and the STRING databases. The results are summarized in Table 3 where the main pathways showing FDR<0.05 are reported. Functional classes showing the lower FDR values include metabolic pathways, oxidative phosphorylation, and neurodegenerative diseases (Parkinson's Disease, Huntington's Disease, Alzheimer's Disease), confirming the effectiveness of the newly developed methods not only in terms of sensitivity in the detection of single up- or down-regulated proteins, but also looking at the biological meaning of aggregated results.

**Table 3:** List of functional pathways identified by STRING analysis.

Pathways Description	Observed Gene Count (NSAF)	FDR	Observed Gene Count (RSC)	FDR	Observed Gene Count (CBN(P))	FDR	Observed Gene Count (CBN(S))	FDR
**Metabolic pathways**	19	2.62E-09	42	1.10E-09	22	2.30E-06	42	1.51E-10
**Oxidative phosphorylation**	7	0.0037	14	9.68E-09	7	0.000105	12	7.09E-07
**Parkinson's disease**	9	0.00049	14	1.77E-08	8	1.53E-05	12	1.11E-06
**Huntington's disease**	9	0.00156	14	2.78E-07	9	9.82E-06	13	1.24E-06
**Alzheimer's disease**	9	0.00111	13	8.40E-07	7	0.000397	12	3.94E-06
**Calcium signaling pathway**	8	0.00412	7	0.0328	7	0.000566	8	0.0109
**Val, Leu and Ile degradation**	6	0.00049	5	0.00648	3	0.0269	5	0.00578
**cGMP-PKG signaling pathway**	8	0.0037	8	0.0094	5	0.02	7	0.0247
**Fatty acid degradation**	5	0.00194	4	0.0254	3	0.0213	4	0.0223

Statistically significant proteins identified by each method were analyzed by STRING, a bioinformatic tool for the functional clusterization in protein networks; all pathways are reported in descending order of FDR.

## Discussion

Label-Free methods for differential proteomics investigations generate a large amount of data that needs to be normalized, i.e. corrected for instrumental or uncontrollable variations before becoming employable for quantitative purposes. The choice of suitable normalization formula is a not trivial aspect in these approaches, because it might strongly affect the experimental results.

For Spectral Counts based Label-Free approaches, normalization procedure has been assessed in different ways, resorting in some cases to the use of fixed and arbitrary corrective factors, such as in Rsc formula [33], to overcome biases associated with the presence of missing values in the analysis, as occurs in NSAF method [39] lacking of adjustment elements.

In light of these considerations, we decide to test new formulas for SpCs data normalization, in which the corrective factor was not an arbitrary choice of the operator, but strictly and directly defined by an intrinsic property of the system under investigation, such as its complexity.

A new approach defined Complexity Based Normalization (CBN) was introduced for the normalization of Spectral Counts data in differential proteomics experiments to calculate the relative protein Fold Changes. The new formula employed a complexity-based adjustment factor generating two different methods, CBN(P) where f = 1/P and CBN(S) in which f = 1/t with P and t referring to the total number of identified proteins (P) or the total spectral counts in the LC-MS/MS analysis (t), respectively.

The performances in terms of reliability, precision, accuracy, linearity, and sensitivity of the two CBN methods in the analysis of raw data from differential proteomics experiments carried out according to the label-free strategies were evaluated in comparison with existing SpC-based normalization approaches, NSAF and Rsc.

Methods evaluation was carried out by analyzing a set of samples consisting of six different standard proteins spiked in an *E*. *Coli* total tryptic digest matrix and the capability of the methods to quantify both the total matrix and the spiked proteins were monitored. Both CBN(P) and CBN(S) showed very good reproducibility in the analysis of the technical replicates and were able to correctly quantify the unchanged amount of *E*. *Coli* proteins. When the accuracy of all methods was compared, CBN(P) showed the best behavior, while the CBN(S) performance was comparable with the one showed by NSAF and RSC methods; a similar trend has been detected also for unchangeable spiked proteins.

In addition, other differences could be observed between all methods when the coefficient of variation in the quantification of *E*. *Coli* proteins was examined. Although both CBN methods displayed a low dispersion of data and low CV, CBN(P) displayed a better performance than CBN(S) suggesting that the precision is affected by the size of the adjustment factor.

The evidence that the choice of the adjustment factor value might affect performances of SpC-based normalization approaches is confirmed also for NSAF and R_SC_ methods. In fact, NSAF showed low reproducibility greater dispersion of data and very high CV value especially in the quantification of the lowest abundant proteins. This is likely due to the absence of any adjustment factor in this method making NSAF very susceptible to quantitative biases caused by the absence of data. The Rsc method showed low reproducibility in the quantification of lowest abundance proteins in the quantitative analysis of *E*. *Coli* proteome but low coefficient of variation in the quantitation of spiked proteins suggesting again that a fixed adjustment factor "f" might affect the analysis of a large set of data.

Otherwise, all quantitative methods were characterized by a wide linearity concentration-range when changeable spiked proteins were quantified, proving a response directly proportional to the protein amount in a wide concentration range.

When CBNs methods were tested on biological complex samples (protein extracts from the brain of HD mice vs WT), both methods generated reproducible, accurate, sensible, and reliable quantitative data. Their FC trends were always confirmed by western blot and/or MRM validations, suggesting that a complexity-based adjustment factor properly works in the correction of output data, leading to suitable final quantitative measurements of protein expression variations.

However, the two CBN methods performed differently in the Fold Change calculation. Although CBN(P) recognized a lower number of changeable significant proteins, it has proven to be the most reliable method to appreciate minimal statistically significant changes in protein expression levels, since its FC cutoff was the narrowest among those calculated for other methods.

Moreover, when the FC values calculated by all methods for selected proteins were compared with those calculated by western blot and/or MRM, a surprisingly perfect accordance was found solely with FCs derived from CBN(P), suggesting that this method performs as the highest reliable and accurate among all. CBN(S), in most cases, performed more like Rsc and NSAF than CBN(P).

Nevertheless, CBN(S) method showed a higher sensitivity in the detection of statistically relevant FC of low abundant proteins that give rise to a low number of SpCs, such as UQCRQ and hnRNP H. These proteins were statistically significant solely for CBN(S), but their FCs were confirmed by western blot assays with a good accordance.

Differences in performances between CBN(P) and CBN(S) might be due to the dimension of the adjustment factor, which is not negligible, considering that the difference between total SpCs and total proteins is about two orders of magnitude (in mice analysis, total SpCs = 2.45x10^5^ and P = 2860).

Moreover, over-representation analysis of CBNs data carried out by STRING reveals that the most of proteins flows in molecular pathways affected in HD brain tissue and other neurodegenerative model systems, thus confirming the robustness and the biological coherence of data provided by these new methods. Indeed, processes such as energy metabolism [5,48–51], oxidative phosphorylation and mitochondria functionality [52–54], calcium homeostasis [55,56], already known to be deregulated in cortical samples from HD mice, were identified, thus indicating the highest correlation level of functional data.

In conclusion, in this study, we demonstrated how all SpCs normalization methods are strongly affected by the presence/absence or by the value of adjustment factors “f”. The presence of a correction factor allows overcoming the effect of the absence of data in FC calculation and leads to methods with the lowest coefficient of variation. Moreover, the association of the "f" with sample complexity makes the operator free from the choice of the best value for the correction factor.

Our data have shown that CBN(S) and CBN(P) are both a viable alternative to other existing SpC-based quantification methods. Furthermore, both CBN(S) and CBN(P) are two sensitive methods, although each shows a different reactivity: CBN(P) is capable of appreciating small but statistically and biologically significant FC variations in proteins well represented in the proteome; on the contrary, the CBN(S) enhances the differences in levels of protein expression in lower abundant proteins.

Finally, if someone would ask which methods we prefer, our choice falls on CBN(P), because it performs as the most reliable and sensitive “sensor” for FCs. In fact, from our point of view, it is preferable to work on a lower number of data which, however, are the result of more stringent and reliable selection standards, rather than getting lost in a sea of data often difficult to interpret from a biological point of view.

## Supporting information

S1 Fig Median values for the best fitting slopes calculated for each pair of technical replicates including mixture B for all samples analyzed with each normalization method. (PDF)

S2 Fig Images of the SDS-PAGE of mice samples. Three biological replicates cortices of zQ175 and WT mice were loaded and gel slices were cut following the scheme reported.
(PDF)

S3 Fig Images of the entire membranes whose inserts are reported in Fig 5A. Full-length western blots. Technical replicates of HTT, IRGM1, Homer1, OSBPL2, hnRNP H, UQCRQ and Samm50 in zQ175 and WT mice were developed with same antibodies. Technical replicates for SerpinB6 were developed with two different antibodies, as reported below the images.
(PDF)

S1 Table Details of the qualitative and quantitative mass spectrometric analysis of standard mixtures spiked in standard E. Coli proteome. In sheet 1, raw data generated by MaxQuant following the bioinformatic analysis for protein identification and quantification are reported. The processed protein groups applying each SpCs method normalization formulas are disclosed in sheets 2, 3, 4, and 5.
(XLS)

S2 Table Linear regression best-fit value referred to MixA. Included is Slope, Y-intercept, and X-intercept calculated by NSAF, RSC, CBN(P), and CBN(S) methods.
(XLS)

S3 Table Fold Change value (FC) and expected Fold Change (exp FC) value by NSAF, RSC, CBN(P) and CBN(S) methods. Included is Fold Change value (FC) and expected Fold Change (exp FC) value of standard proteins (HBA, HBB, BSA, ADH, PYG, and ENO) measured and/or calculated in B/A, C/A, D/A, E/A, C/B, D/B, E/B, D/C, E/C and E/C mixture by using NSAF, RSC, CBN(P), CBN(S).
(XLS)

S4 Table Details of the qualitative and quantitative mass spectrometric analysis of zQ175 and Wild-Type mouse HD model. In sheet 1, raw data generated by MaxQuant following the bioinformatic analysis for protein identification and quantification are reported. The processed protein groups applying each SpCs method normalization formulas are disclosed in sheets 2, 3, 4, and 5.
(XLS)

S5 Table Statistically significant identified proteins. In the table are reported the FCs and p-values of statistically significant identified proteins (p value<0.05) and FDR (<5%). Protein names, gene names, Uniprot codes and peptides are also indicated as headers.
(XLSX)

S6 Table Summary of MRM results. In the table are reported the FCs values of proteins validated by Multiple Reaction Monitoring (MRM). Run, protein names, peptide sequences, Collision Energy, m/z of precursor ions, m/z of fragment ions, total transition area, peptide FC, and averaged and normalized FC for each protein.
(XLSX)
