# Supplementary material for: New label-free methods for protein relative quantification applied to the investigation of an animal model of Huntington Disease
Source: PLoS One. 2020 Sep 4;15(9):e0238037. doi: 10.1371/journal.pone.0238037 (PMC7473538; doi:10.1371/journal.pone.0238037)
Supplement: S1 Fig — (PDF) [file pone.0238037.s001.pdf]

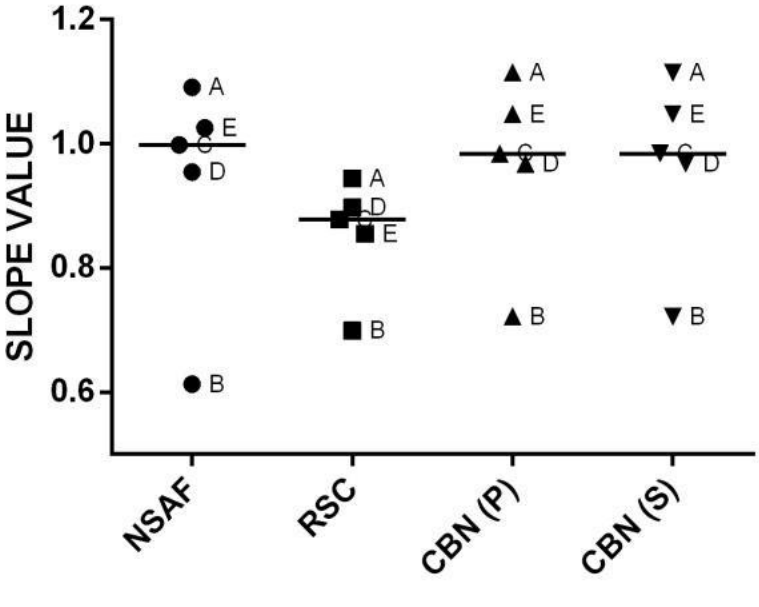

Supplementary Figure S1: Median values for the best fitting slopes calculated for each pair of technical replicates including mixture B for all samples analyzed with each normalization method.
