# Supplementary material for: New label-free methods for protein relative quantification applied to the investigation of an animal model of Huntington Disease
Source: PLoS One. 2020 Sep 4;15(9):e0238037. doi: 10.1371/journal.pone.0238037 (PMC7473538; doi:10.1371/journal.pone.0238037)
Supplement: S2 Fig — Three biological replicates cortices of zQ175 and WT mice were loaded and gel slices were cut following the scheme reported. (PDF) [file pone.0238037.s002.pdf]

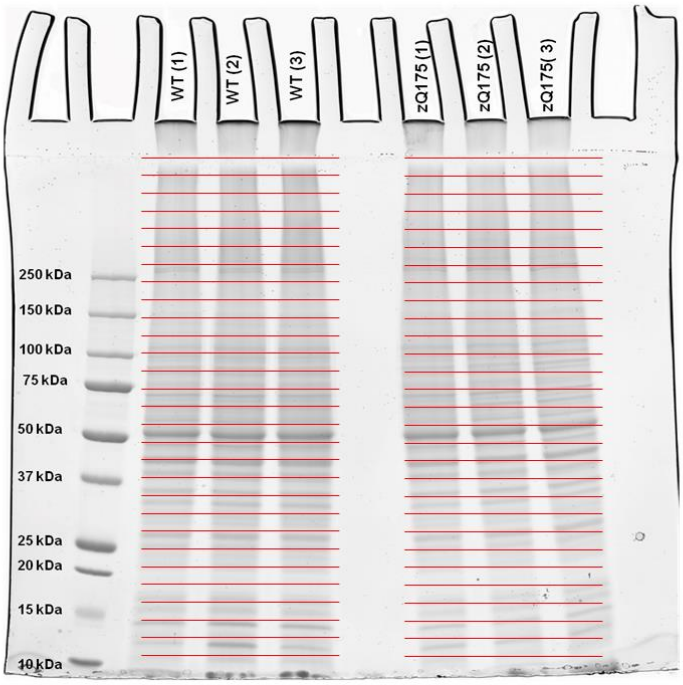

Supplementary Figure S2: Images of the SDS-PAGE of mice samples. Three biological replicates cortices of zQ175 and WT mice were loaded and gel slices were cut following the scheme reported.
