# Supplementary material for: New label-free methods for protein relative quantification applied to the investigation of an animal model of Huntington Disease
Source: PLoS One. 2020 Sep 4;15(9):e0238037. doi: 10.1371/journal.pone.0238037 (PMC7473538; doi:10.1371/journal.pone.0238037)
Supplement: S3 Fig — Full-length western blots. Technical replicates of HTT, IRGM1, Homer1, OSBPL2, hnRNP H, UQCRQ and Samm50 in zQ175 and WT mice were developed with same antibodies. Technical replicates for SerpinB6 were developed with two different antibodies, as reported below the images. (PDF) [file pone.0238037.s003.pdf]

Original and uncropped western blot images

HTT

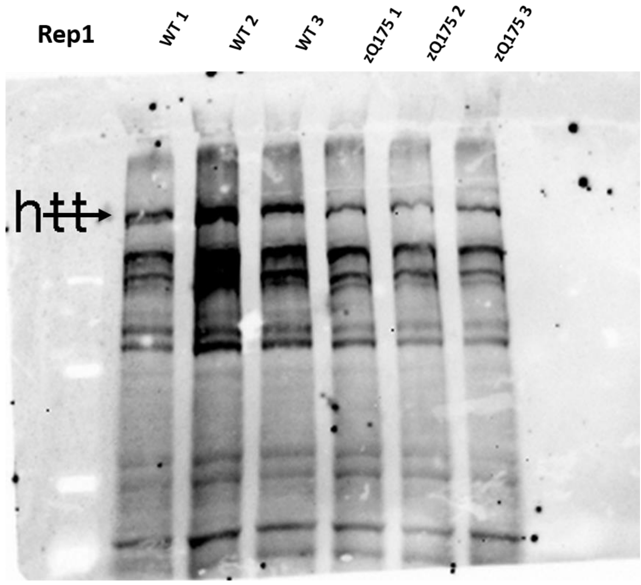

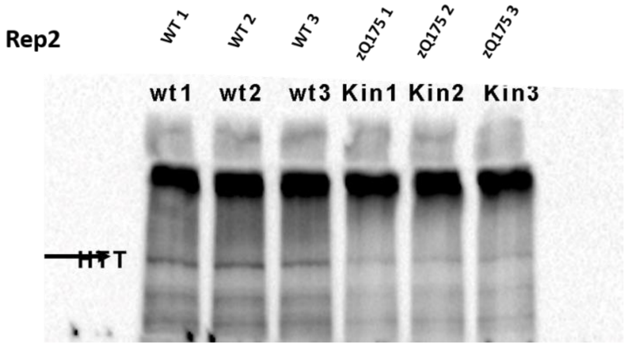

SerpinB6

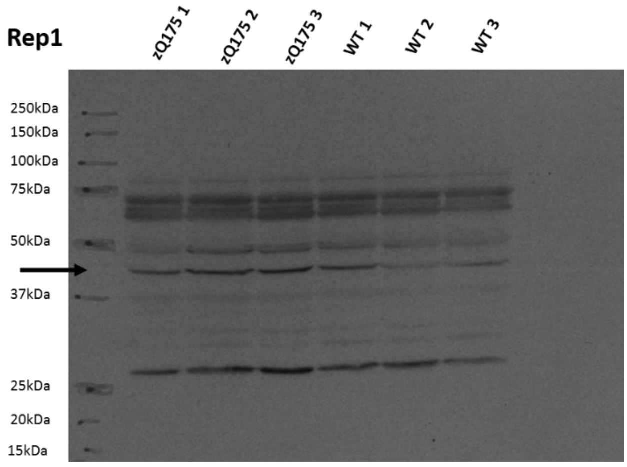

Abcam ab233229

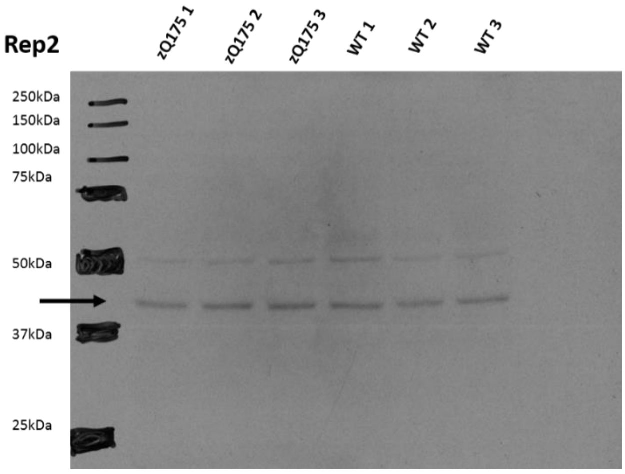

Proteintech 14962-1-AP

IRGM1

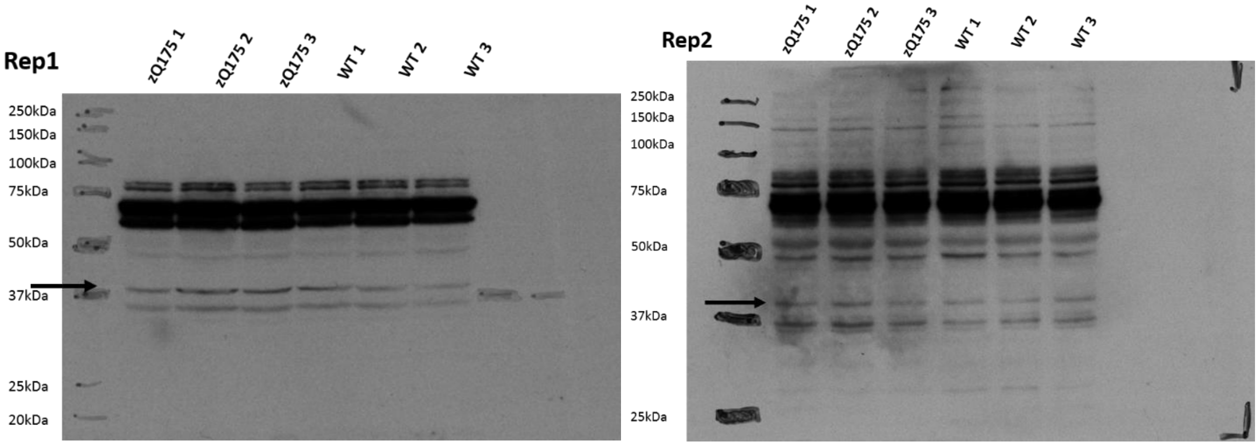

Homer1

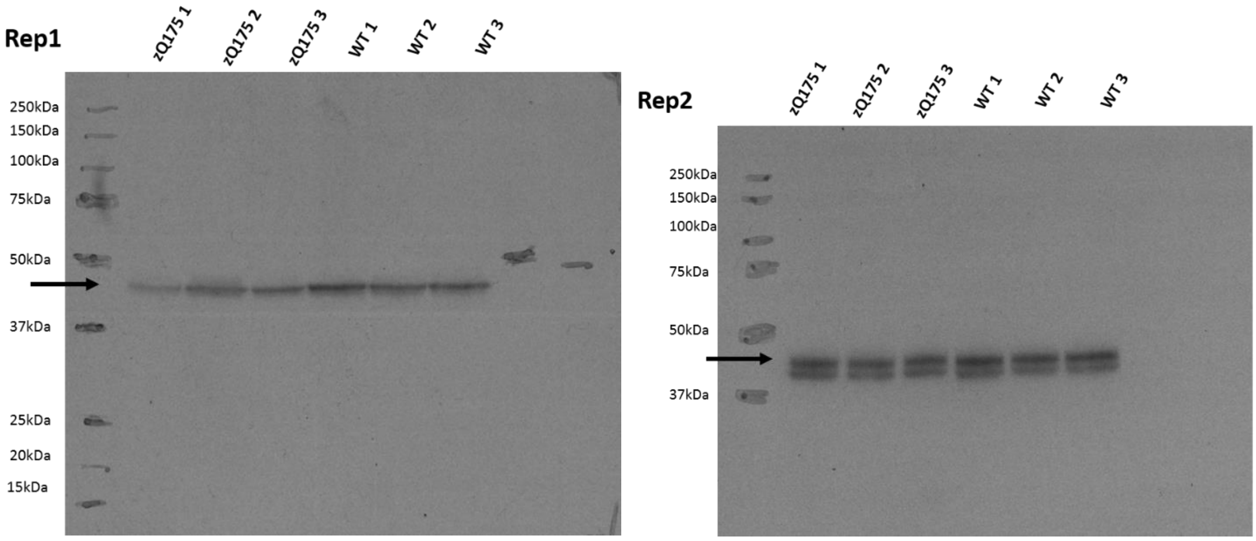

OSBPL2

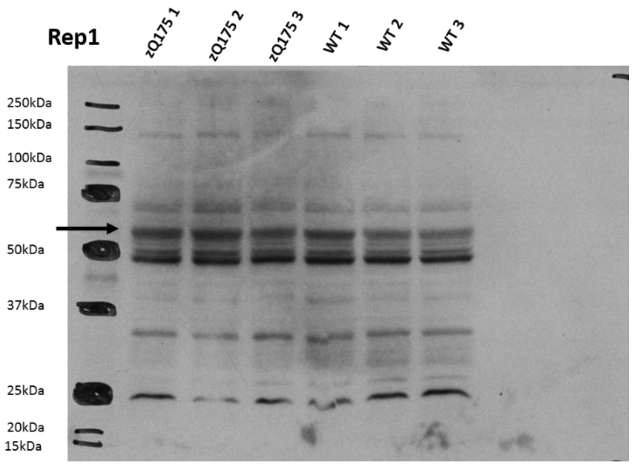

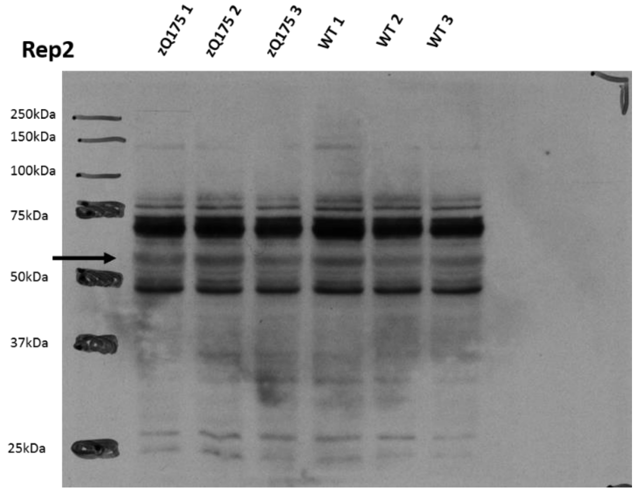

hnRNP H

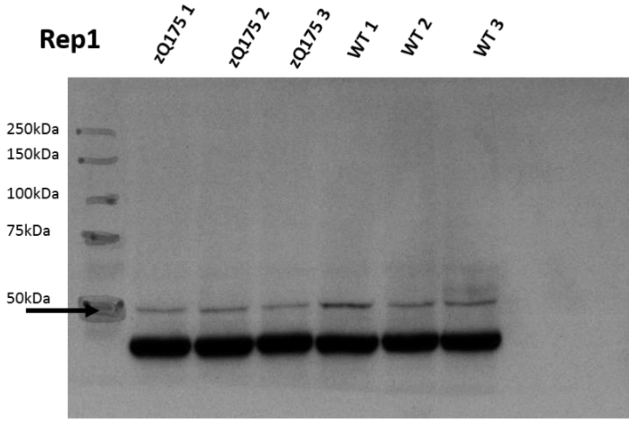

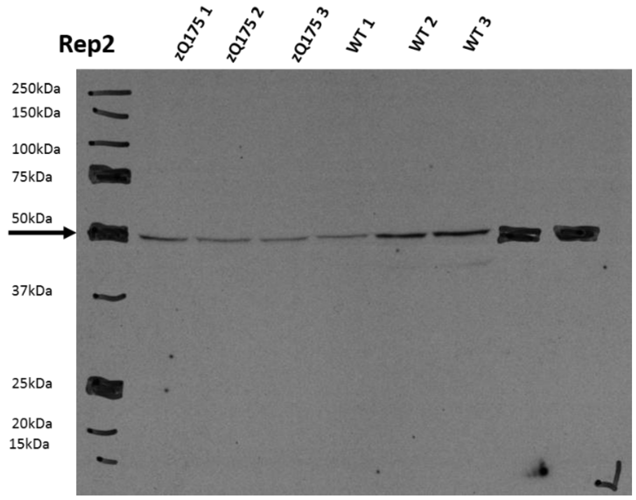

UQCRQ

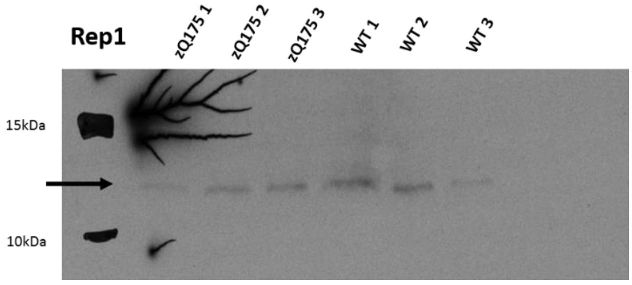

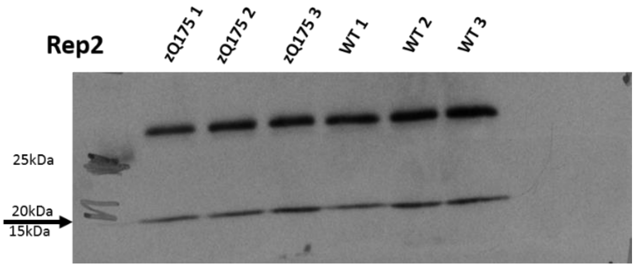

(only the upper part of membrane was developed)

SAMM50

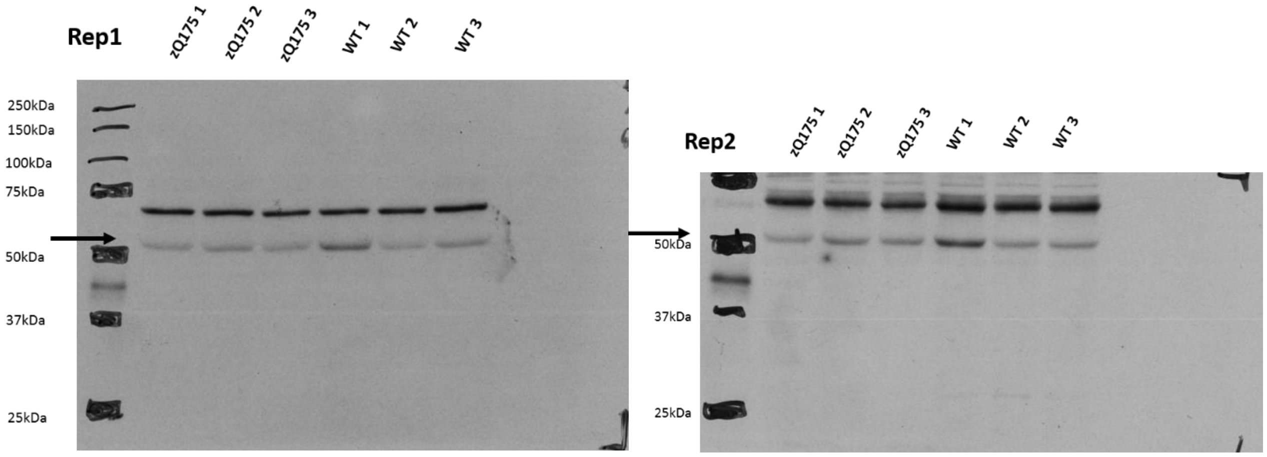

Supplementary Figure S3: Images of the entire membranes whose inserts are reported in Figure 5A. Full-length western blots. Technical replicates of HTT, IRGM1, Homer1, OSBPL2, hnRNP H, UQCRQ and Samm50 in zQ175 and WT mice were developed with same antibodies. Technical replicates for SerpinB6 were developed with two different antibodies, as reported below the images.
